# Breathing lessons: Tor tackles the mitochondria

**DOI:** 10.18632/aging.100013

**Published:** 2009-01-16

**Authors:** Toren Finkel

**Affiliations:** Translational Medicine Branch, National Institutes of Health, Bethesda, MD 20892, USA

The study of Pan and Shadel published in the premier issue of* Aging* further extends our understanding of the growing connection between the TOR pathway and mitochondrial function [1]. Interest in the TOR pathway for the aging community was initially spurred by a flurry of reports suggesting that in various model organisms, inhibition of this pathway could trigger lifespan extension [2-5]. In yeast, the TOR pathway regulates a number of diverse biological outcomes. For instance, treatment of *S. cerevisiae* with rapamycin, a highly specific TOR inhibitor, triggers cell cycle arrest, glycogen accumulation, increased autophagy, a global reduction in protein synthesis and sporulation. Adding to the complexity are the observations that in yeast as well as in mammalian cells, the TOR kinase exists in two separate multiprotein complexes. These complexes designated TORC1 and TORC2 have different biological functions as well as different sensitivities to agents such as rapamycin. Further complicating the matter, in mammals there is a single TOR gene that functions in both the TORC1 and TORC2 complexes, while in *S. cerevisiae *there are two distinct TOR kinase genes.

How does a reduction in TOR signaling lead to lifespan extension? Most evidence suggests that the TOR pathway is intimately linked to the sensing of nutrient status. In a simplified sense, TOR signaling is active when nutrients are abundant and inhibited during periods when food is scarce. Such observations have suggested a potential link between TOR activity and other well know strategies such as caloric restriction wherein limited food availability results in lifespan extension. While such links are on one level satisfying, the exact molecular connection between TOR activity and lifespan remains incompletely understood. It is in this context that the work of the Shadel laboratory is quite illuminating [1,6].

As mentioned above, yeast have two TOR kinase genes. While Tor2p deletion is lethal, yeast without Tor1p are viable. Interestingly, these tor1Δ yeast strains are not only capable of surviving but actually have an increased chronological lifespan. Others have also observed that in yeast a decrease in TOR signaling resulted in lifespan extension, although these previous studies have implicated alterations in the stress resistance as the cause of this lifespan extension [5]. In contrast, Shadel and colleagues have previously provided evidence that the increase in chronological life span seen in the tor1Δ yeast strains was intimately connected to a Tor-dependent regulation of mitochondrial respiration [6]. Their data suggested that in yeast, mitochondrial respiratory capacity and ROS production was both sensed and regulated by the TOR pathway.

In the current study, these past results linking yeast TOR to mitochondrial function have been significantly extended. A more detailed analysis of tor1Δ yeast strains have been performed especially with regard to the mitochondrial proteome. This new data suggests inhibition of TOR signaling results in an increase in the amount of mitochondrial oxidative phosphorylation (OXPHOS) subunits. This increase occurs at both the transcriptional and translational levels and involves both nuclear-encoded as well as mitochondrial-encoded subunits. Interestingly, this increase in OXPHOS subunits is not accompanied by an increase in the number of mitochondria, leading the authors to conclude that the net result is an increase in the density of OXPHOS subunits per mitochondria. How such increased density leads to an increase in respiration is not entirely clear, although it is conceivable that more cytochrome elements per mitochondria in turn leads to more overall mitochondrial respiration. This would imply that under basal conditions, the number of cytochrome chains is rate limiting for respiration. Alternatively, it may be that the density of cytochromes in turn influences the formation of higher-order mitochondrial ‘supercomplexes'. These supercomplexes are known to contain multiple individual electron transport components and their formation and function are just beginning to be analyzed in detail [7,8].

Through proteomic analysis, the new study of Shadel and colleagues revealed that TOR inhibition led not only to an increase in OXPHOS components, but also to the increase in a number of other proteins that localize to the mitochondria. One particularly interesting upregulated protein is Yhb1p, a protein previously implicated in the detoxification of nitric oxide. While there are no previous links between TOR activity and NO biology, there is an extensive literature suggesting that nitric oxide can regulate mitochondrial function [9,10]. Furthermore, mouse models have demonstrated a prominent role for NO in mediating the increase in mitochondrial number observed in the setting of caloric restriction [11,12]. Given the known role of the TOR pathway in potentially mediating the lifespan extending effects of low nutrients, this new proteomic connection to nitric oxide homeostasis is particularly intriguing.

Where do these current results leave us with regard to mammalian aging and the role of TOR in regulating mitochondrial metabolism? Accumulating evidence suggests that mTOR can also regulate mitochondrial number and function in mammalian cells [13,14]. Similarly, proteomic analysis of human T cells treated with rapamycin has demonstrated alteration in OXPHOS components including cytochrome c oxidase and ATP synthase [15]. These same electron transport components were also observed to be altered by Shadel and colleagues in their yeast system. Nonetheless, while TOR activity seems to be important in regulating mitochondrial function in both systems, the emerging data suggest that in yeast, TOR inhibition activates mitochondrial function. In contrast, similar inter-ventions in mammalian cells appear to reduce mitochondrial function. These observed differences in the direction of TOR regulation of mitochondrial activity complicates any straightforward unifying hypothesis regarding how increased or decrease in TOR activity might alter lifespan in both yeast and mammals. It is important to realize however, that when grown in glucose media, yeast cells preferentially metabolize this six carbon sugar to ethanol. It is only when the media becomes depleted of fermentable carbon that yeast cells undergo what as known as the diauxic shift, and begin to metabolize ethanol through an oxygen and mitochondrial dependent pathway. In contrast, under resting conditions, mammalian cells are usually much more heavily dependent on mitochondrial respiration to meet their ongoing energetic needs. Thus, the role of basal mitochondrial respiration is very different in yeast versus mammalian cells. Understanding and exploring these differences will undoubtedly provide important insight into the growing interconnection of TOR, mitochondria and the rate of living.
